# Exploratory analysis of critical event phases and the impact of team size and performance levels in 24 hour ultra cycling

**DOI:** 10.1371/journal.pone.0321944

**Published:** 2025-07-08

**Authors:** Carlo Dindorf, Jonas Dully, Lukas Maurer, Stephan Becker, Steven Simon, Eva Bartaguiz, Michael Fröhlich

**Affiliations:** Department of Sports Science, RPTU University of Kaiserslautern-Landau, Kaiserslautern, Rhineland-Palatinate, Germany; Ningbo University, CHINA

## Abstract

This study explores the complex dynamics of rank-order stability by analyzing the current-to-final rank difference (CFRD), a metric that provides a dynamic view of rank fluctuations and their impact on the final ranking. This approach enables the identification of critical event phases that significantly influence the final rank. Specifically, we examine how varying team sizes and performance levels shape temporal trends in CFRD during 24-hour cycling races. A comprehensive dataset covering four consecutive years (2019–2022) of a 24-hour cycling race on a 17.9 km repetitive driven road track, encompassing diverse team sizes (solo rider, or teams of 4 and 10), is used. The results indicate significant interactions between time, team size, and performance F(18.04) = 1.74; p = 0.03). As the race progresses, the final standings become progressively more predictable. Solo riders exhibit the least clarity in their final standing throughout the race. In contrast, larger teams achieve a clearer indication of their final ranking earlier in the race. Medium-performance teams, especially solo riders, show lower clarity in their final standing across the race duration, whereas high- and low-performance teams tend to exhibit more predictable outcomes at earlier stages of the race. Overall, this study advances our understanding of endurance team cycling, and offers valuable insights for strategic decision-making and race optimization.

## Introduction

Ultra-endurance team cycling races, including 24-hour events, represent a unique and demanding domain within competitive cycling [1,2]. These events have become remarkably popular, with growing numbers of competitions and athletes [3].

Insofar as these are not raced by solo riders, the dynamics of these races extend beyond individual performance metrics as they hinge on the collective efforts of teams comprising various numbers of riders. Ultra cycling team events require collaborative effort, as teams strive to complete as many laps as possible within a designated timeframe. This format is often characterized by continuous relay-style cycling, with teammates taking turns after completing laps. Given the extended event duration, additional considerations arise, including the need for strategic breaks and designated sleep times.

Current cycling science focuses on physiological [4–6] and biomechanical [4,7–9] parameters, nutrition intake [10,11], and aerodynamic improvements [4,12]. Of course, these are the most important for improving performance and efficiency, and therefore, rank better in contests. However, all performance-relevant aspects in ultra team cycling events remain under-represented in the extant literature. For example, studies have shown that teammate attributes influence individual performance during cycling tours [13]. In ultra-endurance team cycling events, the role of teammates is especially crucial, as substitutions are needed to maintain a high average speed over the entire event. Therefore, additional performance-related aspects, besides the aforementioned conventional ones, come into play in ultra cycling.

Studies have attempted to predict lap-by-lap performance and finishing times [14], and the differences in lap-times [15] but only for short-to-mid-distance races. A current study examined the impact of varying speeds and positioning characteristics throughout triathlon team relays. Findings highlight the significance of the latter stages of the race in determining final standings. [16]. Further, this study showed that positioning after the second swim has great influence in the prediction of the final position. Another study by Kholkine *et al*. [17] attempted to predict cycling race outcomes based on the rankings of other races. However, similar studies on ultra team cycling competitions are especially underrepresented in the literature [18,19]. This may be related to the availability of the database. Few studies use public data for (ultra-endurance) races [15,20–23]. In endurance team cycling, competition results are typically presented as lap-times per team in 24-hour races (e.g., Rad am Ring (https://radamring.de/)). Based on this, each team’s ranking position per lap can be derived. In view races, additional information such as the rider completing each lap is provided (e.g., Kaindorf Ultra Rad Challenge (https://www.ultraradchallenge.com/)). Hence, we need approaches to extract meaningful insights from the available data. Therefore, central to the study of endurance team cycling is the analysis of ranks using adequate metrics which can contribute to a comprehensive understanding of the complex dynamics at play in this sport. Such analyses can shed light on the factors that influence success, and strategic decisions that teams make to achieve stability or adapt to changing circumstances during these demanding races.

**Fig 1:**
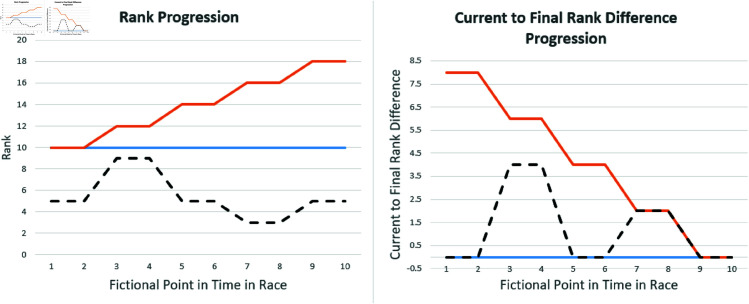
Visualization of the absolute Current-to-final Rank Difference (CFRD) for three different team ranking scenarios (orange, blue, black). Regarding rank stability throughout a race, high stability suggests a relative constant rank throughout the race (blue line). Then, the CFRD is low, as the start and end ranks align. High rank instability throughout the race, however, results in high fluctuations in the CFRD (black line).

Renfree and Casado [24] explain that endurance races are complex systems and even though physiological and psychological factors have a great influence in the race, the race itself may be more complex than only the output of each single athlete. Consequently, they advocate for the development of novel methodologies to thoroughly analyze race dynamics. In line, this study proposes the current-to-final rank difference (CFRD) metric to understand how teams perform over the duration of an endurance team cycling race. The metric offers a dynamic perspective on rank fluctuations and their relation to the final ranking. It quantifies the absolute disparity between a team’s current rank during the race and its final rank after race completion. This metric captures a team’s progression throughout the race, focusing on rank changes rather than the final rank itself (see Fig 1). Therefore, it allows us to detect critical phases of the event that substantially determine the final rank. Critical phases, in this context, refer to specific periods during the race characterized by substantial fluctuations in rank, indicating moments where teams experience significant challenges or opportunities that directly influence their ultimate standing in the race. To the best of our knowledge, a comparable study objective was only investigated in cyclo-cross races by Bossi *et al*. [18]. The authors revealed a progressively positive correlation between intermediate standings and overall rankings. Renfree *et al*. [25] examined pacing strategies across different performance levels and have shown differences between top and bottom finishers. A separate study examining factors influencing race performance, and consequently, finishing positions over an extended time span has only been conducted by Becker *et al*. [26]; however, the authors focused on a season in car races.

As the dynamics of ultra cycling races extend beyond individual performance (except for solo riders), understanding how different team sizes impact CFRD becomes imperative. Further, different team performance levels might affect race dynamics and critical phases, thereby determining the final rank. For example, low-performing teams may face challenges that introduce unpredictability, thus affecting competition dynamics. To the best of our knowledge, neither aspect has been considered in research on ultra-endurance team cycling races.

Here, we seek to unravel the intricacies of CFRD in ultra-endurance team cycling races, focusing on the influence of team size and performance level. Our overarching question is whether (a) team size (solo riders, or teams of 4 and 10; hereinafter, T1, T4, and T10) and performance level over time intervals of a 24-hour cycling race affect CFRD. Subsequently, we analyze (b) temporal differences in critical phases of the event that substantially determine the final rank across teams and performance levels, and how (c) team sizes and (d) performance levels influence CFRD during a 24-hour cycling race.

The current study refrains from formulating explicit hypotheses, following the exploratory approach inspired by Bortz and Döring [27]. This decision is due to the existing, largely unclear state of research, which does not allow for the establishment of hypotheses regarding the mentioned questions. Additionally, this approach ensures that the exploration of further research avenues is not unduly restricted from the outset.

## Materials and methods

### Race data and data collection

**Fig 2:**
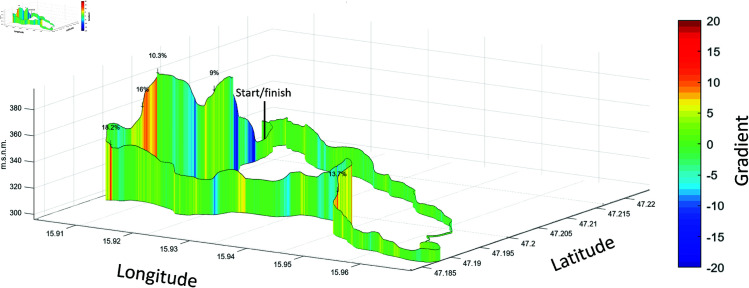
Race track profile. This figure is made with the GPXprofile add on [28] in MATLAB (MathWorks Inc., Natick, Massachusetts, United States). m.s.n.m. = height above mean sea level.

Data from a 24-hour cycling race performed over multiple years were publicly available and collected from myraceresults.com (race results AG, Pfinztal, Germany) for T1, T4, and T10 from the years 2019-2023 of the Kaindorf Ultra Rad Challenge. The event under scope is a ultra-endurance cycling event in Kaindorf, Austria. Solo riders, teams of four or ten riders were allowed to participate with variable team sizes up to the maximal number of riders in the respective category. The goal was to ride as many rounds of the described track as possible in 24 hours. The riders did not have to ride non-stop. Each team was limited to one rider on track at a time. To our knowledge, there are no rules regarding how often each rider must ride or the changing frequencies of athletes. Crucially, the riders were not obligated to switch after completing each lap; they had the flexibility to perform multiple consecutive laps. The races started at 8 pm and ended at 8 pm the following day. All these races took place around mid-July (see Table 1 for detailed information), except in 2020, when the race was delayed to early August due to the COVID-19 pandemic. The track, which was driven in rounds, is 17.9 km long, asphalted, and has an altitude difference of 185 m per lap (see Fig 2). Detailed information about the weather can be found in Table 1.

**Table 1:** Descriptive statistics of the weather data for each year. Weather data did show 0 mm of precipitation for any of the races.

Date	Mean temperature [°C]	Max temperature [°C]	Min temperature [°C]
19.07.-20.07.2019	22.1	28.1	17.7
07.08.-08.08.2020	21.6	29.0	12.0
23.07.-24.07.2021	20.5	26.5	16.1
22.07.-23.07.2022	26.5	30.2	22.3
**Date**	**Mean wind speed [km/h]**	**Max wind speed [km/h]**	**Mean humidity [%]**
19.07-20.07.2019	4.62	7.4	61.29
07.08-08.08.2020	5.03	13	57.26
23.07.-24.07.2021	4.22	5.5	66.13
22.07.-23.07.2022	5.34	7.4	53.4

We merged data from the four consecutive years from 2019 to 2022. This consolidation aimed to enhance the sample size to improve the generalizability and factor size, thereby facilitating more robust statistical analysis. Initially, the data included information on female athletes; however, owing to the small sample size, these data were excluded from the planned analysis. Mixed teams (male/female) were also present, which were counted as male teams by the organizer when at least one rider was male. The corresponding teams were not excluded.

Since some solo riders participated in multiple years, we addressed the potential violation of the independence assumption by randomly selecting only one race entry per individual. In contrast, we treated each team’s participation as an independent observation, even if the same team name appeared across multiple years. This approach was justified by the greater year-to-year variability in team performance compared to solo riders, driven by key factors such as training status, team composition, strategic decisions, and rider order. These fluctuations introduce substantial changes that reduce dependency between observations, thereby supporting the assumption of independence. To ensure comparability between years, we normalized the ranks (see the data preprocessing subsection). Table 2 provides an overview of the descriptive characteristics of the race years for each team size of the final data used.

**Table 2:** Descriptive statistics of the races per year and in total by different team sizes. Note that the maximum number of laps documented is 50.

Date	Team size	Number of teams	Mean number of maximal laps	Mean time for first lap [s]
19.07.-20.07.2019	1	50	28.18±9.09	1877.46±242.93
	4	33	41.42±7.04	1737.21±305.79
	10	16	44.38±4.69	1711.00±285.43
07.08.-08.08.2020	1	43	27.65±8.42	2033.21±225.61
	4	20	42.60±4.72	1822.80±193.88
	10	13	43.92±4.75	1835.15±183.11
23.07.-24.07.2021	1	56	26.57±9.26	2096.79±344.89
	4	27	43.63±4.71	1745.67±197.05
	10	14	43.50±5.18	1775.71±218.20
22.07.-23.07.2022	1	70	24.51±9.93	2065.76±353.80
	4	19	40.26±5.55	1804.68±171.20
	10	14	43.86±4.28	1725.50±197.50
**Total**	1	219	26.49±9.40	2024.31±315.54
	4	146	42.04±5.87	1769.76±234.16
	10	76	43.93±4.74	1758.77±226.87

Table 1 details the weather conditions for each year collected from Meteostat (Meteostat, Friedberg, Germany), and time and date (Time and Date AS, Stavanger, Norway). Notably, the data across different years demonstrate a high degree of similarity in terms of weather patterns and performance.

### Data preprocessing

For each team denoted as *a* (a=1,2,…,A), where *A* is the total number of teams, data was present for each lap (crossing of the finish line), denoted as *l* (l=1,2,…,L), where *L* is the total number of laps per team. This data comprises the lap times *t*_*a*,*l*_ for each team *a* and lap *l*. Based on the sum of riding time for each team at every lap crossing (cumulative riding sum), the rank *R*_*a*,*l*_ is given for each team *a* and lap *l*.

A maximum of 50 laps were recorded and documented in the official race results. As specific lap-by-lap data beyond the 50th lap were unavailable and could not be accurately reconstructed, the results of the 50th lap were employed as the final race results for the few teams surpassing the 50-lap threshold. This affected 14 of the 461 teams that were able to complete more than 50 laps (median: 52 laps for affected teams; maximum: 54 laps completed).

Given the diverse participant numbers across multiple events, a direct comparison of ranks was difficult. For instance, a shift of 10 ranks in a race with 100 participants might appear relatively significant, whereas the same change in a race with 1000 participants could be perceived as relatively minor. To address this issue, we employed the ‘sklearn’ ‘MinMaxScaler’ [29] to normalize ranks based on the total number of participants in each event. This scaling procedure transformed all ranks into a standardized range between 1 (indicating the best rank) and 100 (indicating the lowest rank), thereby facilitating a more meaningful comparison across events.

The Current-to-final Rank Difference (CFRD) is determined based on this as the absolute difference between the team’s rank in the current lap and its final rank upon race completion $R_{a,l_{\text{final}}}$ as follows:


$${\text{CFRD}}_{a,l}=\left|R_{a,l}-R_{a,l_{\text{final}}}\right|$$

For a more intuitive understanding of the metric, please refer to Fig 1.

For every team size, denoted with *i* (i=1,4,10), we categorized the participating teams seperately for each year into three levels (low, medium, high) [23] based on the normalized final ranks with the goal of obtaining three groups of performance levels for every team size of approximately the same size. The performance levels are denoted with *j* (j=1(low),2(medium),3(high)).

During this 24-hour period, teams may cover different numbers of laps. However, laps are not the primary focus of a 24-hour race; rather, the objective is to cover the maximum distance possible within the 24-hour timeframe. For better comparability among teams, the use of total riding time proved to be more effective as it remained consistent for all teams. Hence, our approach involved organizing the information into 2-hour time intervals over the 24-hour race period denoted as *b* (b=1,2,…,B), where *B* is 12 due to the 2-hour binning. These bins were characterized by the median normalized absolute rank difference, which was calculated from the lap-wise data within each specific time interval.

However, data were not necessarily available to calculate median values for 2-hour time intervals for every team, as teams might take a break of over 2 hours or terminate riding within the 24-hour time frame. Therefore, the data for teams that terminated their ride before the official race end were considered until the last calculable time interval. Subsequent time intervals for the CFRD were set to 0 because the final round determined their final position, which resulted in a stable rank for the subsequent race duration. Missing values owing to ranks held during periods of temporary inactivity were filled with the next valid CFRD time interval value.

### Statistics and further calculations

The lowest and highest 5% of values were clipped to reduce the influence of outliers, constraining them within the 5th and 95th percentiles. For our statistical analysis, we employed the Analysis of Variance (ANOVA), a robust method with ability to handle categorical and repeated measures data, as present in our study. Its utility lies in its capacity to efficiently compare means across multiple groups, controlling for Type I error and offering interpretable insights into both the presence and magnitude of group differences [30,31]. To examine variations among various team sizes and performance levels (considered between-subjects factors) throughout a 24-hour race (treated as a within-subjects factor), a mixed ANOVA was conducted (for a detailed explanation of how the statistical model operates and calculates effect sizes and error terms, please refer to [32]). Due to the non-fulfillment of assumptions such as homoscedasticity and comparable covariances, a nonparametric version was employed utilizing the R package nparLD, reporting ANOVA-type statistics [33]. It is important to note that, for the test, the denominator degrees of freedom were set to infinity. As a result, only the numerator degrees of freedom (df) of the central F distribution and the corresponding p-value are reported.

Next, we explored the simple main effects of team size and performance level segregated by other factors in the presence of interactions. We opted for a Welch ANOVA owing to its increased robustness in handling unequal variances among groups. Subsequently, post hoc tests were conducted considering the potential alpha error accumulation, and pairwise comparisons were performed using the Games-Howell method. Both analyses were performed using the Python library pingouin [34].

Finally, to analyze the simple main effects of the within-subjects factor time, repeated measures ANOVA with Greenhouse-Geisser correction was applied if sphericity was violated. The assumption of normality was checked using Shapiro-Wilk tests and Q-Q plots, and normality can be assumed. Post-hoc analyses were conducted using paired t-tests with Bonferroni correction, which was also facilitated by the Python library pingouin [34].

As further exploratory analysis, we investigated the lap time variations between the different team sizes and performance level. Lap time variations were quantified by calculating the absolute time difference between consecutive laps, then aggregating the median values for each team or solo rider. Given the non-normal distribution of the data, statistical comparisons were performed using the Mann-Whitney U test with Bonferroni correction, implemented via the SciPy Python library, to assess significant differences. [35].

The significance level for all analyses was set to 0.05. Visualizations were performed in Python using Seaborn [36].

## Results

**Fig 3:**
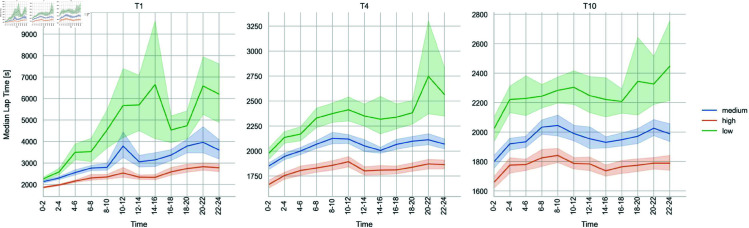
Median lap-times [s] for each time interval by different team sizes and performance level.

Fig 3 shows the median lap-time for each time interval, team size, and performance level. A clear trend of increasing lap times over the course of the race is evident. Notably, the time interval between 6 and 12 hours (corresponding to 12 AM to 8 AM local time) exhibits an initial, temporary increase in lap times for most team sizes and performance levels, potentially due to rest periods and a reduction in riding speed.

### Main interaction effects between team-size, performance level and time (a)

**Fig 4:**
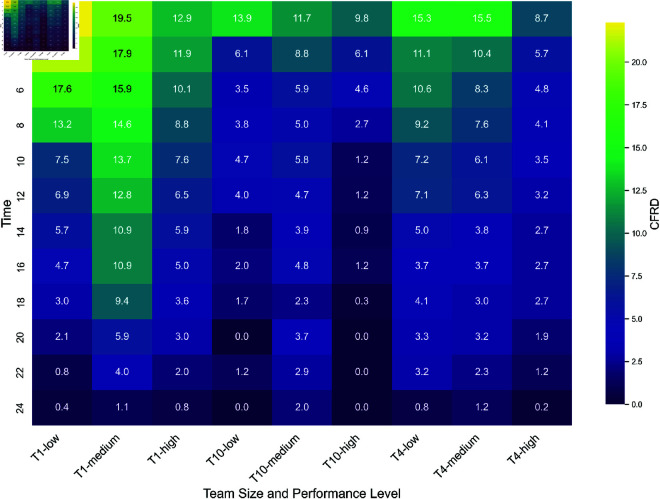
Heatmap of the mean Current-to-final Rank Differences (CFRD) separate for each team size and performance level across the time intervals of the events.

**Fig 5:**
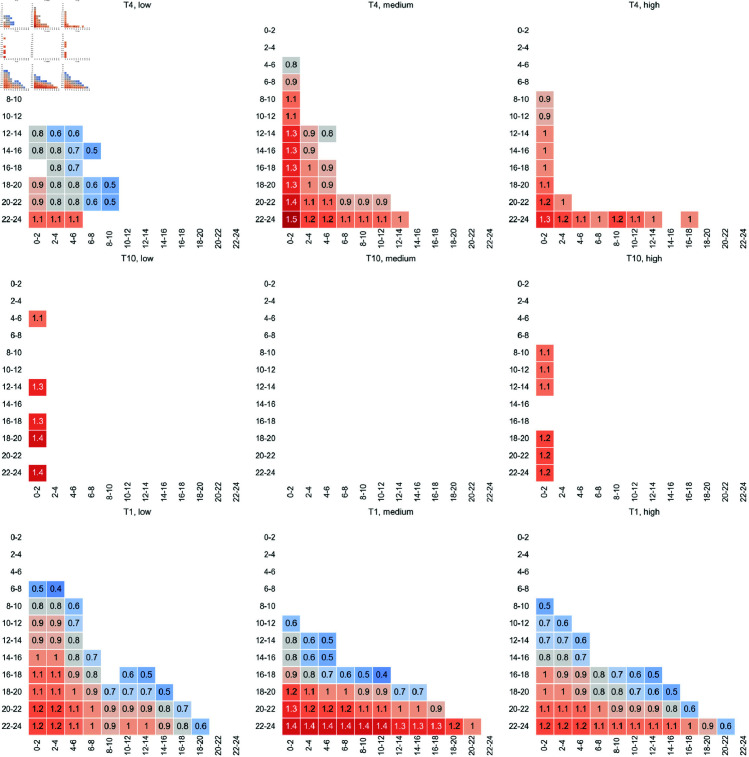
This figure presents Cohen’s d effect size for pairwise comparisons of the time intervals, segregated by team size and their corresponding performance levels. Only statistically significant pairwise effects (p < 0.05) are illustrated. A high Cohen’s d value indicates a substantial effect, emphasizing significant differences between the respective time intervals. The upper triangles are omitted to enhance visual clarity.

**Fig 6:**
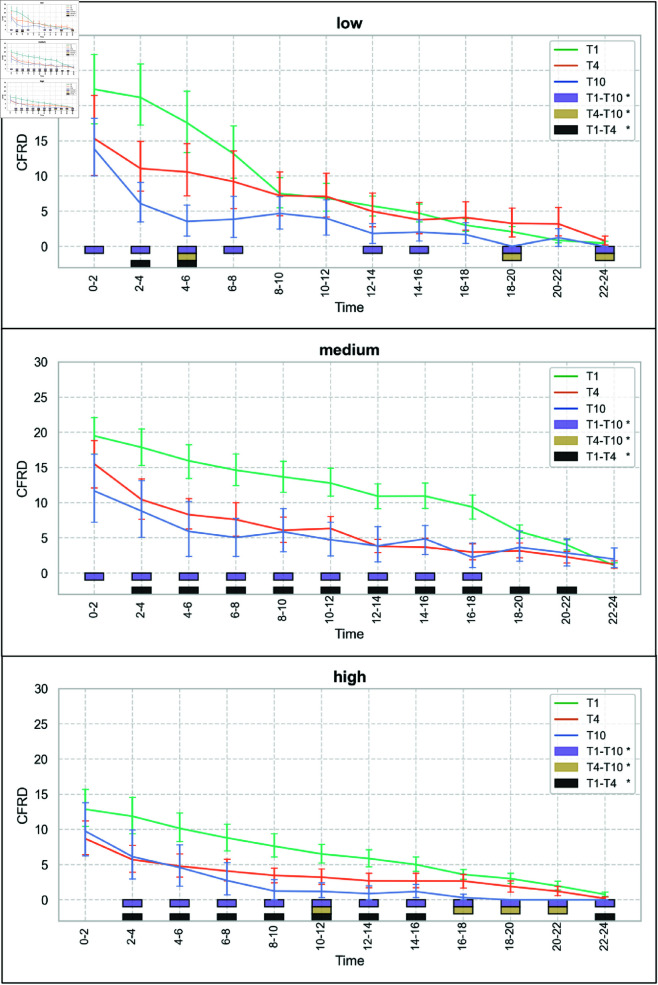
Mean Current-to-final Rank Difference (CFRD) for each performance level and team size over the time intervals of the 24-hour cycling races. Error bars represent 95% confidence intervals. The boxes at the lower end of the plots represent significant pairwise differences (p < 0.05) revealed during post-hoc analysis. To clarify, when referring to "T1-T10" as presented in the legend, a significant pairwise difference between T1 (solo riders) and T10 (teams of ten) is indicated. Similarly, "T4" denotes teams of four.

**Fig 7:**
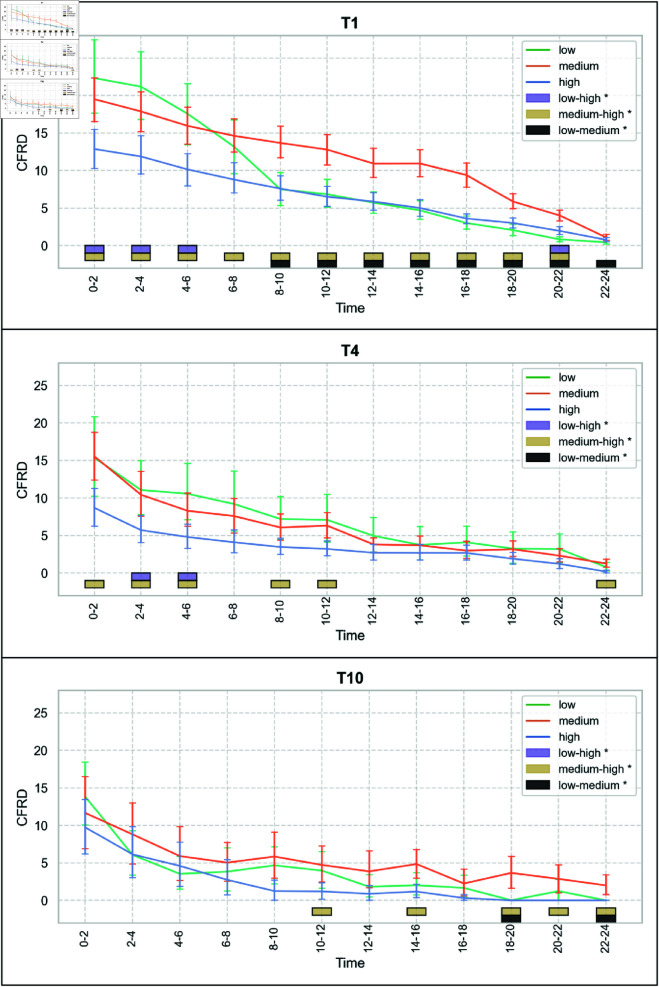
Mean Current-to-final Rank Difference (CFRD) for each team size and performance level over the time intervals of the 24-hour cycling race. Error bars represent 95% confidence intervals. The boxes at the lower end of the plots represent significant pairwise differences (p < 0.05) revealed during post-hoc analysis.

Time, team size, and performance exhibit a statistically significant interaction (a), *F*(18.04) = 1.72, *p* = 0.03. An overview of the mean CFRD seperately for each team size and performance level over the time intervals of the events is visualized in Fig 4. The mean CFRD over the course of the 24-hour races, categorized by team size (distinct lines for performance level), is shown in Fig 6 and categorized by performance level (distinct lines for team size) in Fig 7. Visually, CFRD decreases for all team sizes and performance levels throughout the race. A statistical analysis of the differences between the time intervals for each team size and performance level is shown in Fig 5. We observe distinct patterns for the time interval differences across various teams and performance configurations. Based on the interaction effect between all factors, the following results are separated by the factors.

### Simple main effects of the within-subjects factor time (b)

In summary, T10 shows the fewest differences between time intervals. For the medium performance level, no significant differences across time intervals are observed. In medium- and low-performance teams, only the first time interval (0–2 hours) differs from approximately half of all time intervals throughout the 24-hour period.

In contrast, T1 exhibits the most differences between time intervals. Across all three performance levels, only the initial time intervals show no significant differences in CFRD, specifically from 0–2 to 4–6 hours for low-performance teams and from 0–2 to 6–8 hours for medium- and high-performance teams. Beyond this early phase, subsequent time intervals frequently show significant differences from one another throughout the race. This indicates that CFRD remains relatively stable during the initial phase but then progressively declines, leading to significant differences compared to earlier time intervals. Notably, for the medium performance level, the effect sizes for the differences of the final time interval (22–24 hours) are higher than those observed in both low- and high-performance levels.

In high-performance T4 teams, significant differences are mainly observed between the first time interval (0–2 hours) and later time intervals from 8–10 to 20–22 hours, as well as between the final interval (22–24 hours) and earlier intervals from 0–2 to 18–20 hours, excluding the 14–16 hour interval. In contrast, low- and medium-performance T4 teams also exhibit differences between the initial and concluding phases, though fewer than in T1 teams. Additionally, effect sizes are smaller for low-performance T4 teams. Across all performance levels in T4, the early race time intervals do not significantly differ from each other in terms of CFRD. For low-performance teams, no differences are observed between 0–2 and 10–12 hours. In medium-performance teams, no differences occur between 0–2 and 2–4 hours, while in high-performance teams, no differences are found between 0–2 and 6–8 hours.

### Simple main effects between-subjects factor team size (c)

Separate Welch ANOVAs for each performance level (low, medium, and high) and the time intervals during the 24-hour race reveal significant differences across team sizes. Post-hoc tests are employed for significant Welch ANOVAs, emphasizing the specific time intervals during the race where significant differences between team sizes are observed (see Fig 6).

At the high-performance level, no significant differences in CFRD are observed among team sizes during the initial 0–2 hour interval. However, from that point until the race’s conclusion, T1 consistently maintains a significantly higher CFRD compared to T10. Additionally, from the 2–4 hour interval through the 14–16 hour interval, T1 exhibits a higher CFRD than T4. In the 10–12 hour interval, as well as from 16–18 to 20–22 hours, T4 demonstrates a higher CFRD compared to T10.

At the medium-performance level, T1 significantly differs from T10 from the start of the race up to the 16–18 hour interval, consistently displaying a higher CFRD. Furthermore, from the 2–4 hour interval through the 20–22 hour interval, T1 also maintains a higher CFRD compared to T4.

At the low-performance level, differences between team sizes are less pronounced throughout the race. In the early stages (0–2 to 8–10 hour intervals), T1 exhibits a significantly higher CFRD than T10. Similarly, during the 2–4 and 4–6 hour intervals, T1 has a higher CFRD compared to T4. Notably, in the 4–6 hour interval, all team sizes differ from each other. Beyond this point, differences between team sizes diminish. For a comprehensive breakdown, please refer to Fig 6.

### Simple main effects between-subjects factor performance (d)

Separate Welch ANOVAs for each team size and time intervals reveal significant differences among the low, medium, and high performance levels. Post-hoc tests emphasize the specific time intervals during the race where the performance levels significantly differ (see Fig 7). Overvall it is noticeable that most differences between the performance level are observed for T1.

For T1, notable disparities emerge between the high and medium performance levels from the race start up to the 20–22 hour interval, with the medium level consistently exhibiting a higher CFRD than the high-performance level. In the initial three time intervals (0–2 to 4–6 hours), the low and high performance levels also differ, with lower-performing teams showing a higher CFRD during this phase. However, after the 4–6 hour interval, the gap between low and high performance levels diminishes, and their CFRD values no longer differ significantly. From the 8–10 hour interval until the race concludes, significant differences persist between the low and medium performance levels, with medium-performing teams maintaining a higher CFRD.

For T4, significant differences between the medium and high performance levels are observed from the start of the race until the 10–12 hour interval, except for the 6–8 hour interval. During this period, the medium-performance teams consistently show a higher CFRD. Additionally, at the 2–4 and 4–6 hour intervals, the low and high performance levels also differ, with lower-performing teams exhibiting a higher CFRD.

For T10, differences between performance levels are less frequent. Significant disparities emerge primarily in the middle of the race, where medium-performance teams show a higher CFRD than high-performance teams, specifically in the 10–12, 14–16, and 18–20 hour intervals, continuing until the race’s conclusion. For a detailed breakdown, please refer to Fig 7.

### Further exploratory results

**Fig 8:**
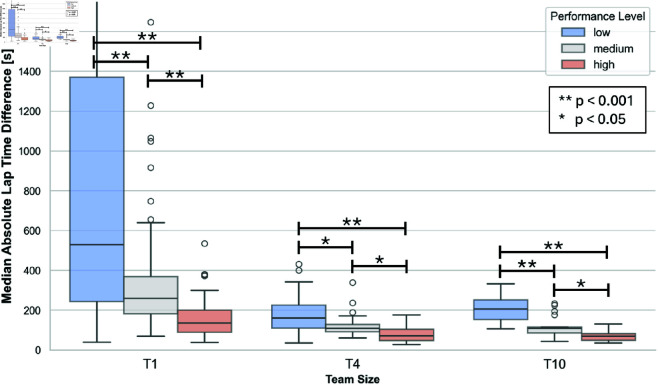
This figure illustrates the median absolute lap time differences for each team size and performance level. Significant differences between performance levels within each team size are indicated. The upper limit of the y-axis has been adjusted to enhance visual clarity.

Within each team size, lap time variations differ across performance levels, with higher-performing teams generally exhibiting lower pace variations (see Fig 8). A comparison of team sizes within each performance level reveals that T1 differs significantly from T4 (U=1901.00, p < 0.001) and T10 (U=1296.50, p < 0.001) in lap time variations at the high-performance level. Similarly, T1 shows differences from T4 (U=1984.00, p < 0.001) and T10 (U=1096.00, p < 0.001) at the low-performance level, as well as from T10 (U=1067.50, p < 0.001) at the medium-performance level. However, lap time variations within each performance level do not differ significantly between T4 and T10 (p > 0.05).

## Discussion

This study addressed the impact of elapsed time, team size, and performance level on CFRD, enhancing the understanding of how these factors interact during extended cycling events. Specifically, we find a substantial interaction effect among team size, performance level, and time on CFRD (a). This suggests that the dynamics of how these variables influence CFRD throughout a race are interrelated rather than being independent.

A higher CFRD suggests that the ongoing phase of the race provides limited insight into the final ranking. Conversely, a lower CFRD implies that during a race, the final ranking is already relatively predictable. Hence, the consistently decreasing CFRD observed across all team sizes and performance levels in 24-hour races suggests enhanced predictability of final rank outcomes as the race unfolds, indicating a tendency for final ranks to converge more closely to the final ranking throughout the event. This aligns with another study of triathlon team relays that showed that especially the latter phases of an endurance competition is relevant for the final positioning of the race [16]. Similarly, previous research has shown that the predictive power of final results increases as competition states get closer to the race conclusion [37].

However, depending on the team size and performance level, unique temporal patterns are found, which can be described as critical phases of the event that substantially determine the final rank (b). For T1, apart from the initial phase, the entire race duration is assoziated with changes in the CFRD, as it gradually decreases over time. However, the final phase plays a particularly crucial role in reducing CFRD, with this effect being most pronounced for medium-performance teams, as reflected by their higher effect sizes. Consequently, the final standings—especially for medium-performance teams—are significantly shaped by the concluding time intervals. Although the effects of group size and performance level were not examined in previous research, related studies highlight that team relays are often decided in the latter stages of the race [16].

T10 shows the fewest differences between time intervals regarding the CFRD and if present only a few for medium- and low-performance teams. This can be interpreted to mean that the final race standing is relatively clear from the beginning of the race, hence the final ranking of the teams is already determined to a large extent at the initial race phase. For T4, the patterns of CFRD changes across time intervals vary the most between performance levels. Apart from the initial and concluding phases of the 24-hour race, CFRD remains relatively stable for high-performance T4 teams. In contrast, low- and medium-performance T4 teams also exhibit differences between the initial and concluding phases, though fewer than in T1 teams.

The findings may be attributed to escalating fatigue [38]. Numerous studies [38–40] have examined fatigue and physiological parameters in ultra-endurance races, showing that the critical role of fatigue emerges around the 5-hour mark in races. Over the course of 24 hours, fatigue sets in, affecting both the physical and mental capacities of the cyclists. Teams of different sizes may experience varying rates of fatigue, affecting their overall performance and rank progression. Note that in this study’s case, the minimum break time for every team rider in T4, based on an average lap speed of 40.28 km/h, results in a total of 80:30 minutes resting time while maintaining a consistent speed and lap-wise exchange rhythm. Even with relatively high-intensity exertion during each subject’s effort over one lap, studies indicate that with this break duration, the impact of fatigue should be minimal [2,41]. For T10, the breaks for each subject are even longer, further diminishing the influence of individual subject fatigue over a 24-hour period. Given the reduced significance of fatigue in determining race outcomes, individual lap speed becomes more crucial and a ranking close to the final rank is established early on.

Based on these considerations, in T1, where riders cannot switch, the impact of fatigue becomes a greater determinant of the final race results. This is because fatigue seems to intensify during the concluding time intervals of the race, also indicated by the increasing lap-times. This results in fatigue-related differentiation in athletes, with a constant and a relatively late pronounced decline in CFRD as observed by the medium performance level. Considering the impact of fatigue on final race results for T1 teams, strategic planning should focus on managing fatigue, especially during the concluding time intervals of the race. Teams may consider adjusting pacing strategies during the later stages of the race to mitigate the impact of fatigue on lap times.

Regarding the nocturnal period an effect on median lap times is observed. However, the temporal characteristics of the CFRD do not show a direct correlation to this nocturnal effect. One potential explanation is that the approach to breaks during the night may have been similar across performance levels. Specifically, the median lap times show a marked increase in lap times for low-performance teams across all team sizes, while the increase is comparatively smaller for high-performance teams. This suggests that low-performance teams may have taken more frequent breaks during the night, while high-performance teams potentially continued with fewer interruptions. The differential performance among high-performance teams during the nighttime may be attributed to variations in individual chronotypes, which can result in divergent performance patterns during nocturnal hours compared to daylight hours [42,43].

The results show significant differences among T1, T4, and T10 team sizes at different performance levels (c). In medium and high-performance teams, T1 consistently differs from T4 and T10 across the race duration, with very few exceptions, maintaining the highest CFRD until the race concludes. This is also reflected in the lap time variations, where T1 teams show significantly greater variations compared to T4 and T10 teams. As a result, for T1, the final outcome remains the least certain throughout the race when comparing the team sizes.

Slightly diverging characteristics are found for the low-performance level, where differences between team sizes are less pronounced throughout the race. In the early stages T1 exhibits a significantly higher CFRD than T4 and T10. Beyond the 4–6 hour interval differences between team sizes diminish. This may be explained by the significance of individual performance in determining the final ranking, which is lower for T4 and T10 teams owing to the shared workload. By contrast, minor fluctuations in individual performance for T1 can trigger significant rank changes and amplify the disparity between the current and final ranks. Despite the flexibility of T4 and T10 in switching riders, the fewer and less pronounced differences in CFRD among low-performance teams of sizes T1, T4, and T10 throughout the race may hint at specific characteristics associated with low-performance races that are seemingly independent of team size. However, further research is essential to delve deeper into this matter and provide more clarity.

The results show that the performance level within the different team sizes significantly influences the CFRD over the 24-hour race (d). After the initial quarter of the race, the medium performance level offers the least predictive insight into the final ranking for T1 across the time intervals. In contrast, the final results are more discernible for teams with high and low performance levels. Studies show that athletes with higher performance levels tend to choose a pace that they can maintain over a long period of time, with less variation in ultra-endurance events [21–23]. This may explain why higher-performing teams tend to exhibit more predictable outcomes over the course of a 24-hour race. Similarly, studies show that people with lower performance levels are less able to assess their actual performance, and therefore, overestimate or underestimate themselves [21,44]. Pace variations may also be attributed to nutrition [11,45] and nutrition intake [46], substantially affecting performance during prolonged physical activities. Insufficient intake of carbohydrates could lead to not-finishing a race [46] and insufficient protein-intake is associated with central fatigue, leading to decreased psychological performance [47]. Notably, nutrition errors are more prevalent among low-performing athletes [48]. High performance riders have a better understanding of their nutritional needs [49] and therefore, in theory, prevent performance loss based on lack of nutrients. This mirrors with our results, where the CFRD is more stable for high performance athletes.

A potential explanation for the greater uncertainty observed in medium-performance teams compared to their high- and low-performing counterparts could be increased variability in pacing strategies, a higher susceptibility to pacing errors, and more pronounced fluctuations in fatigue management. While high-performance teams likely maintain consistent pacing and effective energy distribution, and low-performance teams may operate at a lower but steadier level, medium-performance teams might experience greater inconsistencies, which result in a higher CFRD for the medium-performance athletes.

In T4 and T10, differences between medium- and high-performance levels emerge at specific time intervals during the race, with medium-performance teams exhibiting slightly higher CFRD values. However, compared to T1, the overall differences in CFRD between performance levels remain relatively minor throughout the race. This is further supported by the finding of the exploratory analysis, where lap time variations within each performance level do not differ significantly between T4 and T10. One possible explanation is that teams of multiple riders can compensate for individual lap time inconsistencies, leading to more stable and predictable performance outcomes. Furthermore, in teams with more members, strategic adjustments and workload distribution may play a more significant role in maintaining performance consistency. This collective adaptation could contribute to reducing CFRD differences across performance levels, as fluctuations in individual pacing are more effectively balanced within the team.

This study has certain limitations. First, data from different years were combined to ensure adequate sample size. Although races in different years appear relatively similar at a descriptive level, some differences may exist, which may affect the CFRD. Next, a few T4 and T10 high-performance teams completed more than 50 laps each. However, our data were limited to no more than 50 laps, introducing a potential bias for high-performance teams in the final time interval, as not all ridden laps were considered. Further, we recognized the presence of T10 with fewer than 10 riders, a factor that was not controlled for in our analysis. Other variables, such as crashes, may have occurred and influenced specific years; however, owing to data constraints, these events could not be thoroughly documented and analyzed. Furthermore, the calculation of 2-hour time intervals through binning might lead to a loss of information, given the discrete nature of the interval set.

It is important to note that this analysis primarily focuses on differences in CFRD related to each team. While variations in CFRD may also be influenced by other teams’ behavior, intra-team dynamics, such as a significant change in ranking due to a break, tend to lead to larger variations compared to inter-team factors. Future research can explore the impacts of specific team strategies, such as sleeping time and rider switches, on CFRD and rank stability within such races. Investigating how team characteristics influence these dynamics, especially with heterogeneous performance levels among team members, can provide valuable insights. Next, a more in-depth analysis can be conducted on the factors contributing to stability or instability in team rankings over the course of a race. Furthermore, considering the reported gender differences [3] offers promising research opportunities. Another compelling research avenue is comparative analyses of alternative race formats, such as 12-hour races, or entirely different race types like mountain bike 24-hour races. This can offer valuable insights into the unique dynamics of 24-hour cycling races and how they differ from other endurance events, contributing to a richer understanding of strategic and performance considerations across various race formats. Additionally, in contrast to our inferential statistical approach, mathematical analysis could serve as a valuable method for future research [50], particularly since mathematical simulations have already yielded promising insights in sports science [51].

## Conclusion

In ultra-endurance cycling races, lap-wise rank changes, determined by team lap times, often represent one of the most accessible data points for analysis. The proposed CFRD methodology emerges as a valuable tool for examining key race phases and the intricate interplay between team size and performance level. This study advances our understanding of CFRD in the context of 24-hour cycling races, with particular emphasis on the roles of elapsed time, team size, and performance level. The findings demonstrate a complex interconnection between these factors. As the race progresses, the final standings become progressively more predictable, with critical race phases exerting a pronounced influence on ultimate rankings. Solo riders exhibit the greatest variability in lap times and the least clarity in their final standing throughout the race. In contrast, larger teams achieve a clearer indication of their final ranking earlier in the race. Medium-performance teams, especially solo riders, show lower clarity in their final standing across the race duration, whereas high- and low-performance teams tend to exhibit more predictable outcomes at earlier stages of the race. The nuanced patterns observed offer valuable insights for both scholars and practitioners, enhancing the understanding of the complex dynamics inherent in ultra-endurance team cycling events. The findings advance insights into endurance team cycling, offering actionable knowledge for strategic decision-making and optimizing race performance.

## Acknowledgments

The authors extend their sincere gratitude to the organizers of the Kaindorf Ultra Rad Challenge for generously granting permission to use the data for scientific research purposes.

## References

[1] Knechtle B, Knechtle P, Rosemann T. No association between skinfold thicknesses and race performance in male ultra-endurance cyclists in a 600 km ultra-cycling marathon. Human Movement. 2009;10(2). doi: 10.2478/v10038-009-0018-y

[2] Bescós R, Rodríguez F-A, Iglesias X, Knechtle B, Benítez A, Marina M, et al. Physiological demands of cyclists during an ultra-endurance relay race: a field study report. Chin J Physiol. 2011;54(5):339–46. 22135913

[3] Baumgartner S, Sousa CV, Nikolaidis PT, Knechtle B. Can the performance gap between women and men be reduced in ultra-cycling?. Int J Environ Res Public Health. 2020;17(7):2521. doi: 10.3390/ijerph17072521 32272640 PMC7177769

[4] Phillips KE, Hopkins WG. Determinants of cycling performance: a review of the dimensions and features regulating performance in elite cycling competitions. Sports Med Open. 2020;6(1):23. doi: 10.1186/s40798-020-00252-z 32495230 PMC7271082

[5] Sharma AP, Bentley DJ, Mejuto G, Etxebarria N. A contemporary variable-power cycling protocol to discriminate race-specific performance ability. Int J Sports Physiol Perform. 2020;15(9):1309–14. doi: 10.1123/ijspp.2019-0558 32315983

[6] Gallo G, Filipas L, Tornaghi M, Garbin M, Codella R, Lovecchio N, et al. Thresholds power profiles and performance in youth road cycling. Int J Sports Physiol Perform. 2021;16(7):1049–51. doi: 10.1123/ijspp.2020-0579 33647878

[7] Jongerius N, Wainwright B, Walker J, Bissas A. The biomechanics of maintaining effective force application across cycling positions. J Biomech. 2022;138:111103. doi: 10.1016/j.jbiomech.2022.111103 35512435

[8] Swart J, Holliday W. Cycling biomechanics optimization-the (R) evolution of bicycle fitting. Curr Sports Med Rep. 2019;18(12):490–6. doi: 10.1249/JSR.0000000000000665 31834181

[9] Bartaguiz E, Dindorf C, Dully J, Becker S, Fröhlich M. Effects of increasing physical load and fatigue on the biomechanics of elite cyclists. Sci J Sport Perform. 2022;2(1):59–69. doi: 10.55860/nbmd9425

[10] Crosby S, Butcher A, McDonald K, Berger N, Bekker PJ, Best R. Menthol mouth rinsing maintains relative power production during three-minute maximal cycling performance in the heat compared to cold water and placebo rinsing. Int J Environ Res Public Health. 2022;19(6):3527. doi: 10.3390/ijerph19063527 35329209 PMC8949398

[11] Papadopoulou SK, Xyla EE, Methenitis S, Feidantsis KG, Kotsis Y, Pagkalos IG, et al. Nutrition strategies before and during ultra-endurance event: a significant gap between science and practice. Scand J Med Sci Sports. 2018;28(3):881–92. doi: 10.1111/sms.13006 29117450

[12] Doan CV, Nguyen CT, V. Vu T, Nguyen VD, Phan PTT. Aerodynamic characteristics calculation and shape design optimization of a prototype vehicle. In: AIP Conference Proceedings, 2021. 020034. doi: 10.1063/5.0068710

[13] Torgler B. “La Grande Boucle”. J Sports Econ. 2007;8(3):317–31. doi: 10.1177/1527002506287657

[14] Fitton B, Symons D. A mathematical model for simulating cycling: applied to track cycling. Sports Eng. 2018;21(4):409–18. doi: 10.1007/s12283-018-0283-0

[15] Mantzios K, Ioannou LG, Panagiotaki Z, Ziaka S, Périard JD, Racinais S, et al. Effects of weather parameters on endurance running performance: discipline-specific analysis of 1258 races. Med Sci Sports Exerc. 2022;54(1):153–61. doi: 10.1249/MSS.0000000000002769 34652333 PMC8677617

[16] Martínez-Sobrino J, Del Cerro JS, González-Ravé JM, Veiga S. Race dynamics in triathlon mixed-team-relay meaningfully changes with the new regulation towards Paris 2024. J Sports Sci Med. 2024;23(2):358–65. doi: 10.52082/jssm.2024.358 38841631 PMC11149078

[17] Kholkine L, Servotte T, de Leeuw A-W, De Schepper T, Hellinckx P, Verdonck T, et al. A learn-to-rank approach for predicting road cycling race outcomes. Front Sports Act Living. 2021;3:714107. doi: 10.3389/fspor.2021.714107 34693282 PMC8527032

[18] Bossi AH, O’Grady C, Ebreo R, Passfield L, Hopker JG. Pacing strategy and tactical positioning during cyclo-cross races. Int J Sports Physiol Perform. 2018;13(4):452–8. doi: 10.1123/ijspp.2017-0183 28872369

[19] Babault N, Paizis C, Trimble M, Trimble DA, Cometti C. Pacing and positioning strategies during an elite fixed-gear cycling criterium. Front Sports Act Living. 2020;2:586568. doi: 10.3389/fspor.2020.586568 33345156 PMC7739637

[20] Weiss K, Sousa CV, Thuany M, Cuk I, Nikolaidis PT, Knechtle B. Differences in pacing during cycling and running in ultra-triathlons - The example of “Swissultra”. Eur Rev Med Pharmacol Sci. 2022;26(14):4959–68. doi: 10.26355/eurrev_202207_29281 35916791

[21] Suter D, Sousa CV, Hill L, Scheer V, Nikolaidis PT, Knechtle B. Even pacing is associated with faster finishing times in ultramarathon distance trail running-the “Ultra-Trail du Mont Blanc” 2008-2019. Int J Environ Res Public Health. 2020;17(19):7074. doi: 10.3390/ijerph17197074 32992625 PMC7578994

[22] Bossi AH, Matta GG, Millet GY, Lima P, Pertence LC, de Lima JP, et al. Pacing strategy during 24-hour ultramarathon-distance running. Int J Sports Physiol Perform. 2017;12(5):590–6. doi: 10.1123/ijspp.2016-0237 27618658

[23] Stjepanovic M, Knechtle B, Weiss K, Nikolaidis PT, Cuk I, Thuany M, et al. Changes in pacing variation with increasing race duration in ultra-triathlon races. Sci Rep. 2023;13(1):3692. doi: 10.1038/s41598-023-30932-1 36878948 PMC9986668

[24] Renfree A, Casado A. Athletic races represent complex systems, and pacing behavior should be viewed as an emergent phenomenon. Front Physiol. 2018;9:1432. doi: 10.3389/fphys.2018.01432 30344496 PMC6182257

[25] Renfree A, Crivoi do Carmo E, Martin L. The influence of performance level, age and gender on pacing strategy during a 100-km ultramarathon. Eur J Sport Sci. 2016;16(4):409–15. doi: 10.1080/17461391.2015.1041061 26034882

[26] Becker BE, Huselid MA. The incentive effects of tournament compensation systems. Administ Sci Quart. 1992;37(2):336. doi: 10.2307/2393228

[27] Bortz J, Döring N. Forschungsmethoden und Evaluation für Human- und Sozialwissenschaftler (Sonderausgabe der 4. Auflage). 2006.

[28] Guepud JL. GPXprofile. 2023. https://de.mathworks.com/matlabcentral/fileexchange/77792-gpxprofile

[29] Pedregosa F, Varoquaux G, Gramfort A, Michel V, Thirion B, Grisel O. Scikit-learn: machine learning in python. J Mach Learn Res. 2011;12:2825–30.

[30] Döring N, Bortz J, Pöschl S. Forschungsmethoden und Evaluation in den Sozial- und Humanwissenschaften: Mit 194 Abbildungen und 167 Tabellen. 5th ed. Heidelberg: Springer Medizin Verlag. 2016.

[31] Murrar S, Brauer M. Mixed model analysis of variance. In: Frey B, editor. The Sage encyclopedia of educational research, measurement, and evaluation. Los Angeles and London and New Delhi and Singapore and Washington, DC and Melbourne: SAGE Reference; 2018.

[32] Burdick RK, Borror CM, Montgomery DC. Design and analysis of gauge R & R studies: Making decisions with confidence intervals in random and mixed ANOVA models. Philadelphia, PA: Society for Industrial and Applied Mathematics. 2005.

[33] Noguchi K, Gel YR, Brunner E, Konietschke F. nparLD: AnRSoftware package for the nonparametric analysis of longitudinal data in factorial experiments. J Stat Soft. 2012;50(12). doi: 10.18637/jss.v050.i12

[34] Vallat R. Pingouin: statistics in Python. JOSS. 2018;3(31):1026. doi: 10.21105/joss.01026

[35] Virtanen P, Gommers R, Oliphant TE, Haberland M, Reddy T, Cournapeau D, et al. SciPy 1.0: fundamental algorithms for scientific computing in Python. Nat Methods. 2020;17(3):261–72. doi: 10.1038/s41592-019-0686-2 32015543 PMC7056644

[36] Waskom M. seaborn: statistical data visualization. JOSS. 2021;6(60):3021. doi: 10.21105/joss.03021

[37] Ley C, van de Wiele T, van Eetvelde H. Ranking soccer teams on basis of their current strength: a comparison of maximum likelihood approaches. http://arxiv.org/pdf/1705.09575

[38] Pokan R, Ocenasek H, Hochgatterer R, Miehl M, Vonbank K, Von Duvillard SP, et al. Myocardial dimensions and hemodynamics during 24-h ultraendurance ergometry. Med Sci Sports Exerc. 2014;46(2):268–75. doi: 10.1249/MSS.0b013e3182a64639 23899887

[39] Dodds P, Schoeller D, Shriver T, Ruby B. Total energy expenditure and ad libitum fluid/nutrient intake during a 24-hour mountain-bike event: a case study. Int J Sports Physiol Perform. 2023;18(5):541–6. doi: 10.1123/ijspp.2022-0310 36931326

[40] Demchak TJ, Linderman JK. Ultra endurance cycling: a field study of human performance during a 12 hour mountain bike race. Med Sci Sports Exercise. 1999;31(Supplement):S107. doi: 10.1097/00005768-199905001-00384

[41] Ainsworth BE, Serfass RC, Leon AS. Effects of recovery duration and blood lactate level on power output during cycling. Can J Appl Physiol. 1993;18(1):19–30. doi: 10.1139/h93-003 8471990

[42] Facer-Childs ER, Boiling S, Balanos GM. The effects of time of day and chronotype on cognitive and physical performance in healthy volunteers. Sports Med Open. 2018;4(1):47. doi: 10.1186/s40798-018-0162-z 30357501 PMC6200828

[43] Rae DE, Stephenson KJ, Roden LC. Factors to consider when assessing diurnal variation in sports performance: the influence of chronotype and habitual training time-of-day. Eur J Appl Physiol. 2015;115(6):1339–49. doi: 10.1007/s00421-015-3109-9 25631930

[44] Fariod M, Olher RR, Sousa CV, Scheer V, Cuk I, Nikolaidis PT, et al. Pacing variation in multistage ultramarathons: internet-based cross-sectional study. JMIR Form Res. 2023;7:e46650. doi: 10.2196/46650 37610796 PMC10483293

[45] Bircher S, Enggist A, Jehle T, Knechtle B. Effects of an extreme endurance race on energy balance and body composition - a case study. J Sports Sci Med. 2006;5(1):154–62. 24198693 PMC3818668

[46] Tiller NB, Roberts JD, Beasley L, Chapman S, Pinto JM, Smith L, et al. International Society of Sports Nutrition Position Stand: nutritional considerations for single-stage ultra-marathon training and racing. J Int Soc Sports Nutr. 2019;16(1):50. doi: 10.1186/s12970-019-0312-9 31699159 PMC6839090

[47] Meeusen R, Watson P. Amino acids and the brain: do they play a role in “central fatigue”?. Int J Sport Nutr Exerc Metab. 2007;17 Suppl:S37-46. doi: 10.1123/ijsnem.17.s1.s37 18577773

[48] Tam R, Gifford JA, Beck KL. Recent developments in the assessment of nutrition knowledge in athletes. Curr Nutr Rep. 2022;11(2):241–52. doi: 10.1007/s13668-022-00397-1 35174474 PMC9174104

[49] Reinhard C, Galloway SDR. Carbohydrate intake practices and determinants of food choices during training in recreational, amateur, and professional endurance athletes: a survey analysis. Front Nutr. 2022;9:862396. doi: 10.3389/fnut.2022.862396 35360695 PMC8963786

[50] Haider JA, Muhammad N. Mathematical analysis of flow passing through a rectangular nozzle. Int J Mod Phys B. 2022;36(26). doi: 10.1142/s0217979222501764

[51] Schumaker RP, Solieman OK, Chen H. Predictive modeling for sports and gaming. Sports data mining. Boston, MA: Springer US. 2010. p. 55–63.

